# Tetramethylpyrazine Protects Retinal Capillary Endothelial Cells (TR-iBRB2) against IL-1β-Induced Nitrative/Oxidative Stress

**DOI:** 10.3390/ijms160921775

**Published:** 2015-09-09

**Authors:** Xue Zhu, Ke Wang, Kai Zhang, Xuhua Tan, Zhifeng Wu, Song Sun, Fanfan Zhou, Ling Zhu

**Affiliations:** 1 Key Laboratory of Nuclear Medicine, Ministry of Health, Jiangsu Key Laboratory of Molecular Nuclear Medicine, Jiangsu Institute of Nuclear Medicine, Wuxi 214063, China; E-Mails: zhuxue@jsinm.org (X.Z.); wangke@jsinm.org (K.W.); zhangkai@jsinm.org (K.Z.); 2 Department of Ophthalmology, Wuxi No. 2 People’s Hospital, Nanjing Medical University, Wuxi 214002, China; E-Mail: doctxh001@gmail.com; 3 Faculty of Pharmacy, University of Sydney, New South Wales 2006, Australia; E-Mail: fanfan.zhou@sydney.edu.au; 4 Save Sight Institute, University of Sydney, New South Wales 2000, Australia; E-Mail: ling.zhu@sydney.edu.au

**Keywords:** tetramethylpyrazine, TR-iBRB2 cells, nitrosative stress, oxidative stress

## Abstract

Blood-retinal barrier (BRB) breakdown is one of the primary causes of diabetic retinopathy (DR). The pro-inflammatory factor interleukin-1β (IL-1β) was reported to be involved in the induction of BRB breakdown during the pathogenesis of DR. In the present study, we investigated the protective effect of tetramethylpyrazine (TMP), a major active component of the traditional herb *Ligusticum chuanxiong*, on IL-1β-induced cell death of the rat retinal capillary endothelial TR-iBRB2 cells. Our results showed that IL-1β-induced cell dysfunction in TR-iBRB2 cells via inducing nitrative/oxidative stress; however, such effect was attenuated with the pre-treatment of TMP. The cellular protective effect of TMP was likely to be mediated through the inhibition of inducible nitric oxide synthase (iNOS) expression and leukostasis as well as suppression of reactive oxygen species (ROS) generation, mitochondrial dysfunction and MAPKs activation. These findings significantly contribute to a better understanding of the protective effect of TMP in DR and form the basis of the therapeutic development of TMP in treating such disease in the future.

## 1. Introduction

Diabetic retinopathy (DR) is a prevalent retinal disease and a leading cause of blindness in diabetic patients worldwide. It is caused by changes in the blood vessels of the retina, in particular blood-retinal barrier (BRB) breakdown [1,2]. BRB is a selective diffusion barrier that isolates the retina from blood, maintains the appropriate milieu for optimal retinal function and excludes potentially harmful stimuli; therefore, it acts as a critical protective barrier [1]. BRB breakdown is considered as an early hallmark of DR, which eventually results in vasogenic edema and neural tissue damage leading to vision loss [3].

Up to date, the cellular mechanisms underlying BRB dysfunction are poorly understood; however, it is known that the altered BRB function is a consequence of the altered permeability of retinal endothelial cells caused by elevated levels of growth factors, cytokines, advanced glycation end products, inflammation, hyperglycemia and/or loss of pericytes [3]. Several inflammatory factors have been reported as the important mediators in the pathogenesis of DR, inhibition of which successfully prevented BRB breakdown and progression of DR in mice [4]. Interleukin-1β (IL-1β) is one of the inflammatory factors shown to induce a number of alterations in the BRB including leukocyte recruitment, increased permeability and changes in endothelial cell morphology. Moreover, recent studies provided evidences to show that increased IL-1β in the retinal endothelial cells contributes to the elevated level of nitric oxide (NO) and caspase-dependent apoptosis [5]. Thus, targeted inhibition of IL-1β-induced endothelium dysfunction may be effective in the prevention of BRB breakdown and in the treatment of DR.

Tetramethylpyrazine (TMP) is one of the major active constituents of the traditional Chinese herbal medicine, *Ligusticum wallichii Franchat* (*chuanxiong*). It has been long and widely used by herbalists in the treatment of neurovascular and cardiovascular diseases in China [6,7,8]. In recent years, TMP has been suggested to treat various retinal diseases especially DR due to its anti-oxidant and anti-inflammatory effects [7,9]. *In vitro* and *in vivo* experiments indicated that TMP exerts its effects mainly through protecting neuronal cells from oxidative stress-induced retinal damage [10,11,12,13]. However, the molecular mechanisms underlying the neuroprotective effect of TMP are still poorly understood.

In this study, we performed *in vitro* experiments for the first time to examine the protective effect of TMP against IL-1β-induced retinal endothelial cell damage in TR-iBRB2 cells. This is a rat retinal endothelial cell line isolated from the inner BRB exhibiting the characteristic properties of such cell barrier [14]. We also investigated the potential molecular mechanisms underlying such effect.

## 2. Results and Discussion

### 2.1. Results

#### 2.1.1. Tetramethylpyrazine (TMP) Reduced IL-1β-Induced Inducible Nitric Oxide Synthase (iNOS) Expression and Reactive Oxygen Species (ROS) Generation

**Figure 1:**
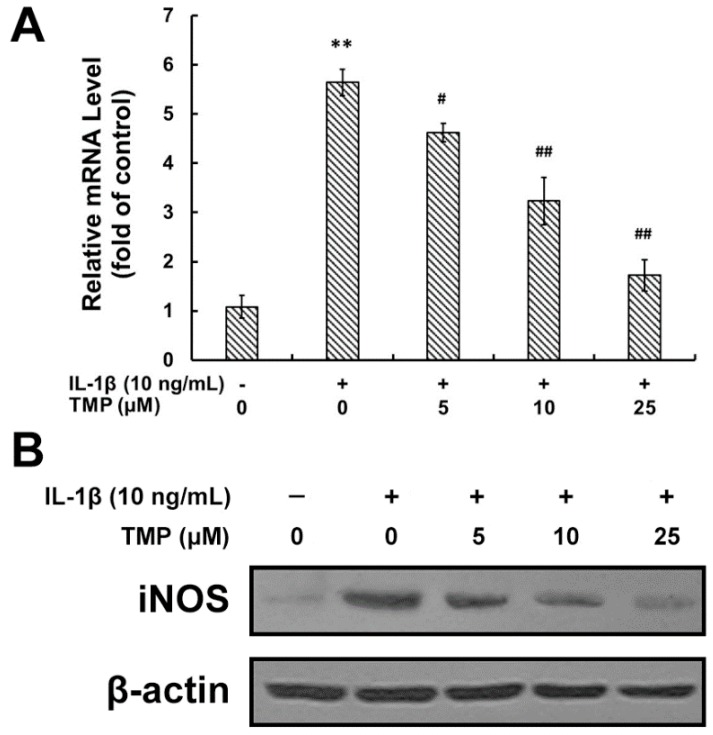

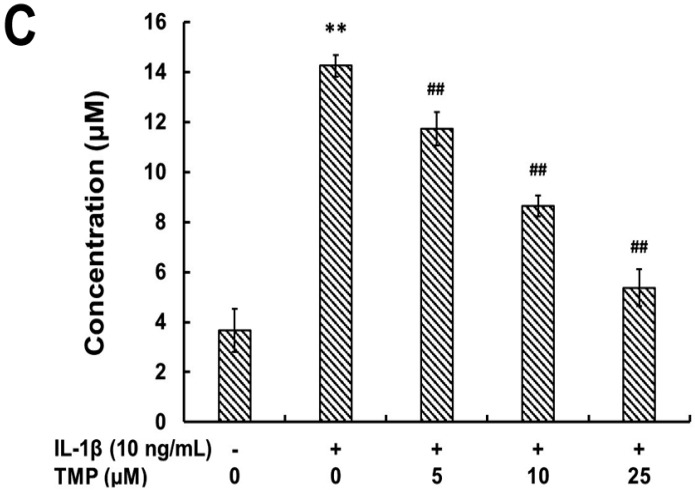
Effect of TMP on IL-1β-induced iNOS and NO expression in TR-iBRB2 cells. After treatment of indicated drugs, the relative mRNA and protein expressions of iNOS were assessed by RT-PCR (**A**) and Western blot analysis (**B**); Nitrite levels of the TR-iBRB2 cells were determined by Griess assay (**C**). All data were expressed as mean ± SD of three experiments and each experiment included triplicate repeats. ** *p* < 0.01 *vs.* control group; *# p* < 0.05; ## *p* < 0.01 *vs.* IL-1β-treated group. iNOS: inducible nitric oxide synthase.

**Figure 2:**
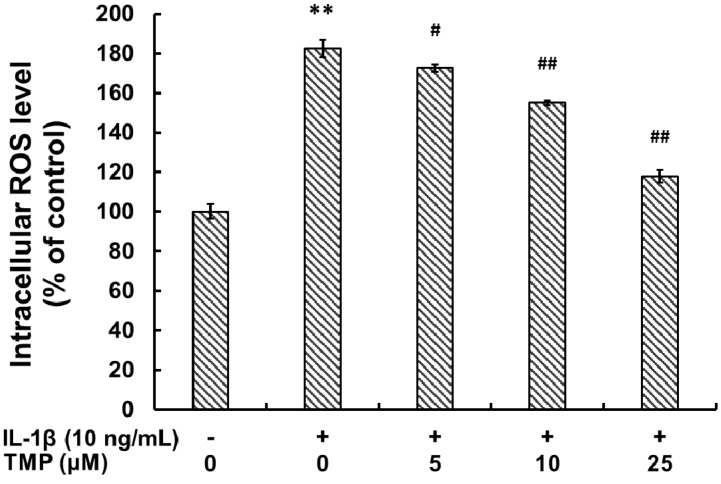
Effect of TMP on IL-1β-induced ROS generation in TR-iBRB2 cells. After treatment of indicated drugs, the intracellular ROS generation in TR-iBRB2 cells was determined by flow cytometry using DCFH-DA staining. All data were expressed as mean ± SD of three experiments and each experiment included triplicate repeats. ** *p* < 0.01 *vs.* control group; *# p* < 0.05; ## *p* < 0.01 *vs.* IL-1β-treated group.

IL-1β is one of the most potent stimuli for the nitrosative/oxidative stress, inferred from the up-regulated expression of iNOS and ROS generation. To investigate the protective effect of TMP, TR-iBRB2 cells were incubated with IL-1β (10 ng/mL) for 6 h after pre-exposed to the indicated concentration of TMP (5–25 μM) for 12 h. The intracellular iNOS levels were analyzed by quantitative real-time PCR and Western blot analysis. As shown in Figure 1A,B, IL-1β stimulation up-regulated iNOS at both mRNA and protein levels in TR-iBRB2 cells; however, such an effect was attenuated with the pre-treatment of TMP. Up-regulation of iNOS resulted in an increased nitrite (NO_2_^−^) level indicating elevated NO production from activated TR-iBRB2 cells, which was also blocked by the pre-treatment of TMP (Figure 1C). Exposure to IL-1β (10 ng/mL) for 6 h significantly up-regulated ROS generation in TR-iBRB2 cells. However, such effect was attenuated with the pre-treatment of TMP at a concentration-dependent manner (Figure 2). Our findings suggested that TMP markedly suppressed IL-1β-induced nitrosative/oxidative stress through inhibiting iNOS expression and ROS generation.

#### 2.1.2. TMP Attenuated IL-1β-Induced Leukocyte Adhesion to TR-iBRB2 Cells and ICAM-1 Expression

**Figure 3:**
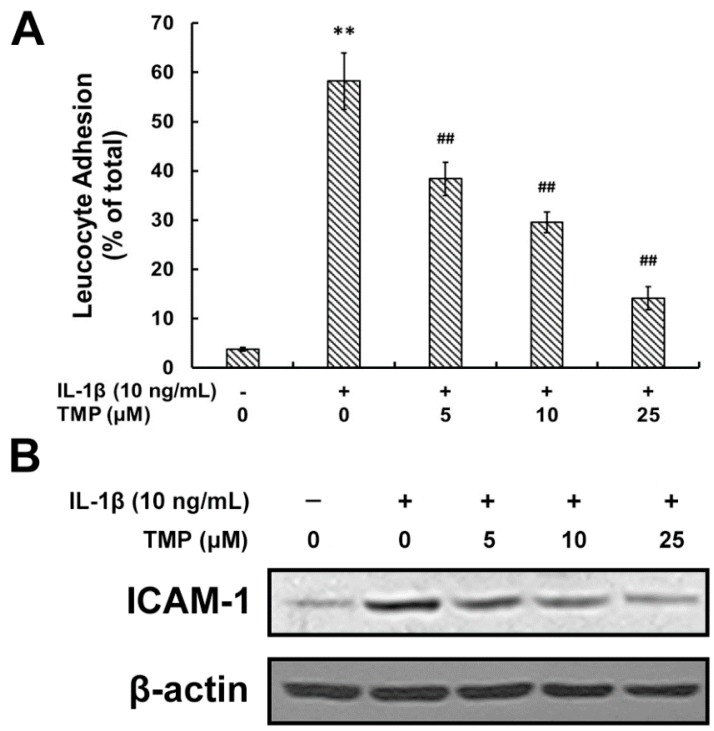
Effect of TMP on IL-1β-induced leukostasis and ICAM-1 expression in TR-iBRB2 cells. After treatment of indicated drugs, leukocyte adhesion to TR-iBRB2 cells was determined as described in Materials and Methods (**A**) and the protein level of ICAM-1 was determined using Western blot analysis (**B**). All data were expressed as mean ± SD of three experiments and each experiment included triplicate repeats. ** p < 0.01 *vs.* control group; *## p* < 0.01 *vs.* IL-1β-treated group.

The rapid generation of NO from inducible (iNOS) source in diabetic retinas contributes to the adhesion of leukocytes to retinal vessels, thus inducing endothelial cell death and ultimately leading to BRB breakdown [15,16,17]. We investigated the effect of TMP on the IL-1β-induced leukocyte adhesion to TR-iBRB2 cells. As shown in Figure 3A, after exposure to IL-1β (10 ng/mL) for 12 h, leukocyte adhesion to TR-iBRB2 cells was significantly increased (58.24% ± 5.74% of total cells); whereas such effect was attenuated with the pre-treatment of TMP (14.12% ± 2.35% of total cells). Furthermore, the involvement of cell adhesion molecules such as ICAM-1 was analyzed in TR-iBRB2 cells treated with various drugs. As shown in Figure 3B, IL-1β (10 ng/mL) stimulation significantly induced a higher expression of ICAM-1 in TR-iBRB2 cells compared to the control. However, the pre-treatment with TMP reversed such effect dose-dependently.

#### 2.1.3. TMP Blocked IL-1β-Induced NF-κB Translocation into the Nucleus in TR-iBRB2 Cells

**Figure 4:**
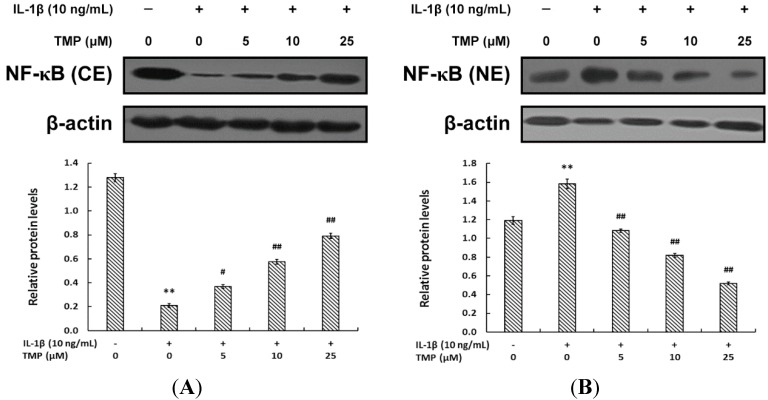
Effect of TMP on IL-1β-induced nuclear translocation in TR-iBRB2 cells. After treatment of indicated drugs, nuclear and cytoplasmic fractions were analyzed for the expression of NF-κB p65 subunit by Western blot analysis. (**A**) Relative protein level of NF-κB p65 subunit in cytoplasm of TR-iBRB2 cells; (**B**) Relative protein level of NF-κB p65 submit in nucleus of TR-iBRB2 cells. All data were expressed as mean ± SD of three experiments and each experiment included triplicate repeats. ** *p* < 0.01 *vs.* control group; *# p* < 0.05, ## *p* < 0.01 *vs.* IL-1β-treated group.

The transcription factor Nuclear Factor κB (NF-κB) modulates the expression of iNOS and other inducible genes such as *COX-2*, *VCAM*-1 and *ICAM*-1 in immune and inflammatory responses. Here we assessed the role of NF-κB in the protective effect of TMP against IL-1β-induced inflammatory response in TR-iBRB2 cells. Cells were pretreated with TMP at concentrations ranging from 0 to 25 μM for 12 h before exposure to IL-1β (10 ng/mL) for 12 h. Our results showed that IL-1β (10 ng/mL) stimulation promoted the translocation of NF-κB from cytoplasm into the nucleus in TR-iBRB2 cells via the measurement of NF-κB p65 subunit expression in cytoplasm and the nucleus. Interestingly, TMP pre-treatment reversed the regulatory effect of IL-1β in a concentration-dependent manner (Figure 4). Our findings suggested TMP could inhibit the nitrosative stress induced by IL-1β through decreasing the activity of NF-κB, thus suppressing the expression of iNOS and ICAM-1.

#### 2.1.4. TMP Attenuated IL-1β-Induced Cellular Injury and Apoptosis in TR-iBRB2 Cells

**Figure 5:**
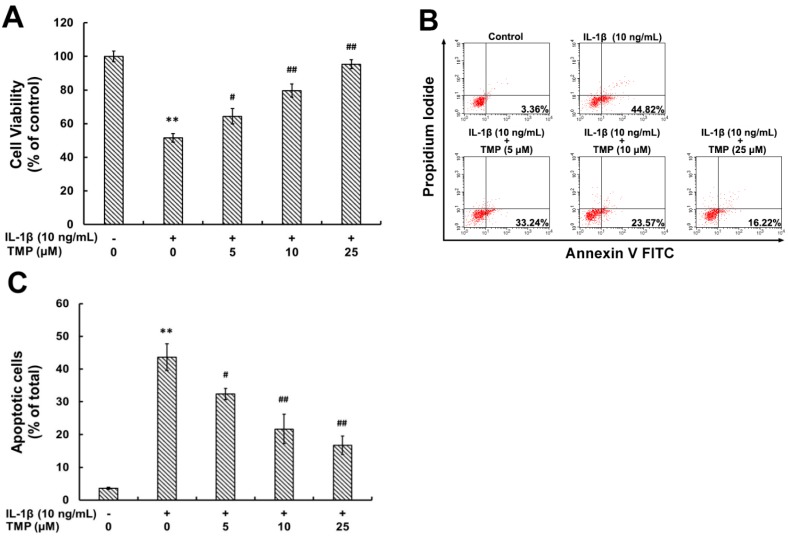
Effect of TMP on IL-1β-induced cell injury and apoptosis in TR-iBRB2 cells. With the treatment of indicated drugs, cell viability was evaluated by MTT assay (**A**) and cell apoptosis was evaluated by the Annexin V-PI dual-staining assay (**B**,**C**). All data were expressed as mean ± SD of three experiments and each experiment included triplicate repeats. ** p < 0.01 *vs.* control group; *# p* < 0.05, *## p* < 0.01 *vs.* IL-1β-treated group.

IL-1β-mediated cell death and apoptosis have also been suggested to contribute to the pathogenesis of DR [5].TR-iBRB2 cells were exposed to IL-1β (10 ng/mL) for 6 h after pretreatment with TMP at various concentrations for 12 h. Our cell viability data showed that IL-1β treatment (10 ng/mL) caused significant death of TR-iBRB2 cells. However, pre-treatment of TMP at various concentrations (5, 10 and 25 μM) markedly reversed the cytotoxic effect of IL-1β (Figure 5A). Furthermore, cell apoptosis was determined by Annexin V-FITC and propidium iodide (PI) double staining using flow cytometry. As showed in Figure 5B,C, IL-1β treatment (10 ng/mL) significantly increased the percentage of early apoptotic cells, while pre-treatment of TMP (25 μM) resulted in a decreased cell apoptosis from 43.62% ± 4.13% to 16.73% ± 2.86%.

#### 2.1.5. TMP Decreased IL-1β-Induced Mitochondrial Dysfunction and Phosphorylation of MAPKs in TR-iBRB2 Cells

**Figure 6:**
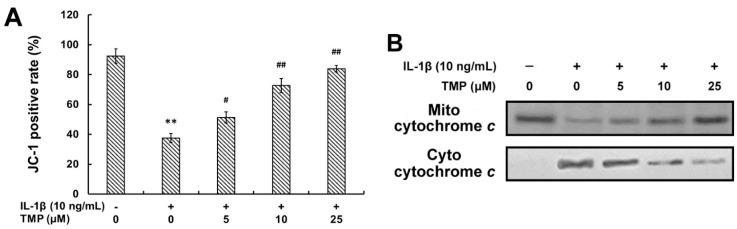
Effects of TMP on IL-1β-induced mitochondrial dysfunction in TR-iBRB2 cells. After treatment of indicated drugs, the level of MMP was determined using the ratio of fluorescence at 590 nM to that at 530 nM (**A**) and the expression of cyto- and mito-cytochrome *c* was determined by Western blot analysis (**B**). All data were expressed as mean ± SD of three experiments. ** *p* < 0.01 *vs.* control group; # *p* < 0.05; ## *p* < 0.01 *vs.* IL-1β-treated group.

**Figure 7:**
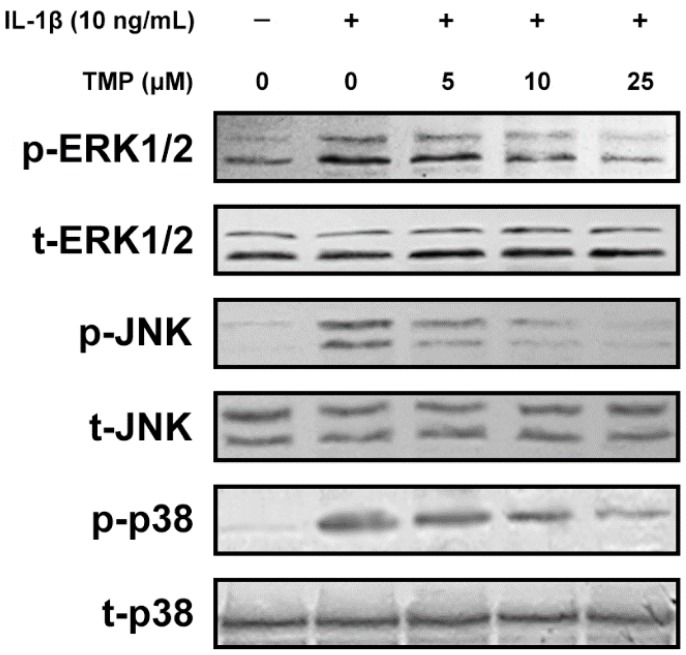
Effect of tetramethylpyrazine (TMP) on IL-1β-induced phosphorylation of MAPKs in TR-iBRB2 cells. With the treatment of indicated drugs, the phosphorylation of ERK1/2, JNK and p38 MAPK was determined by Western blot analysis. All data were representative of three independent experiments.

As the major source of ROS, mitochondria is exposed to high concentration of ROS and therefore, is particularly susceptible to oxidative injury [18]. An excessive amount of ROS can induce mitochondrial permeability transition and the release of cytochrome *c* into the cytoplasm, which is one of the major events of cell apoptosis [19]. After pretreatment with TMP at various concentrations for 12 h, the cells were exposed to IL-1β (10 ng/mL) for 6 h. As shown in Figure 6, IL-1β (10 ng/mL) treatment significantly decreased the level of mitochondrial membrane potential (MMP) and up-regulated the release of cytochrome *c* from mitochondria in TR-iBRB2 cells, while this effect was attenuated by TMP pre-treatment in a concentration-dependent manner. It is known that the Mitogen-activated protein kinases (MAPKs) are involved in both cell growth and death, which can be activated by ROS [20]. As shown in Figure 7, ERK1/2, JNK and p38 MAPK were highly phosphorylated in TR-iBRB2 cells treated with IL-1β alone as compared to that of the control; however, the phosphorylation of these proteins was inhibited by TMP pre-treatment in a concentration-dependent manner. Our findings suggested that TMP inhibited IL-1β-induced oxidative stress mainly through inhibiting ROS generation, mitochondrial dysfunction and MAPKs activation, and thus suppressing cell cytotoxicity and apoptosis.

### 2.2. Discussion

In the present study, we demonstrated for the first time that TMP, a potent antioxidant and anti-inflammatory agent could inhibit IL-1β-induced cytotoxicity in the rodent retinal capillary endothelial TR-iBRB2 cells. Our results suggested that IL-1β-induced endothelial cell injury is associated with iNOS-mediated inflammatory response as well as ROS-regulated mitochondrial dysfunction and apoptosis. All these effects could be attenuated by TMP at low concentrations (5–25 μM).

Because retinal endothelial cells are vulnerable to inflammatory factors, it is generally accepted that retinal vessel inflammation is a primary cause leading to BRB breakdown. IL-1β has been shown to be one of the most potent stimuli for iNOS production and inflammatory responses in such pathological process [5]. Previous studies indicated that TMP could attenuate TNF-α-induced iNOS expression in endothelial cells [21,22]. Consistently, we found that low doses of TMP (5–25 μM) could suppress iNOS expression at both transcriptional and post-transcriptional level in IL-1β-induced TR-iBRB2 cells. Increased level of iNOS further up-regulates ICAM-1 expression at the surface of endothelial cells, when exposed to pro-inflammatory molecules such as IL-1β. Up-regulation of ICAM-1 promotes leukocyte adhesion to endothelial cells, which leads to BRB breakdown in diabetes [23]. Our present study indicated that IL-1β-induced ICAM-1 expression and leukostasis were significantly inhibited by TMP pre-treatment. The increase in leukostasis was also associated with the activation of NF-κB, an important transcription factor involved in inflammatory responses. Usually, NF-κB is located in cytoplasm as an inactive complex bound to its inhibitory factor κB-α (IκB-α). Once activated by inflammatory mediators like IL-1β or TNF-α, NF-κB will be disassociated from IκB-α and translocates into the nucleus to modulate the transcription of its target genes [24]. NF-κB activation is known to induce the adhesion molecules (ICAM-1, VCAM-1 and E-seletin), cytokines, growth factors and iNOS expression [24,25]. NF-κB inhibition has also been found to reduce the chance of leukostasis and BRB breakdown in diabetic rat retinas [26]. Indeed, our study revealed that IL-1β markedly decreased the expression of NF-κB p65 subunit in cytoplasm but increased that in nucleus, which regulatory effect was effectively reversed by TMP pre-treatment in a concentration-dependent manner. Hence, it suggests that attenuating nitrative stress by TMP is critical for IL-1β-induced inflammatory response in TR-iBRB2 cells.

Cytokines induced oxidative stress is another important mechanism underlying the pathogenesis of DR [27]. Elevation of ROS in excess of the buffering capacity and enzymatic activities designed to modulate ROS levels result in a potentially cytotoxic “oxidative stress” [28,29]. Mitochondria are the major source of super oxide, peroxynitrite and hydroxyl radicals; however, they are also vulnerable to oxidative damage. ROS-triggered mitochondrial dysfunction could impair mitochondrial membrane potential, release cytochrome *c* and provoke apoptosis [30]. Our study indicated that the accumulation of intracellular ROS induced by IL-1β led to cellular injury and apoptosis in TR-iBRB2 cells. In addition, these effects were effectively inhibited by TMP pre-treatment. TMP may function as an antioxidant in retinal endothelial cells. Indeed, our data showed that TMP pre-treatment reduced intracellular ROS generation, up-regulated MMP and inhibited cytochrome c release in IL-1β-stimulated TR-iBRB2 cells. Moreover, ROS can act as the second messenger to activate MAPKs, a pathway that is involved in many cellular functions under pathological conditions [31,32]. Our study indicated that TMP remarkably attenuated the elevated phosphorylation of ERK1/2, JNK and p38 MAPK in TR-iBRB2 cells induced by IL-1β. Therefore, TMP could reduce IL-1β-induced oxidative stress and apoptosis through attenuating ROS-mediated mitochondria dysfunction and MAPKs phosphorylation.

**Figure 8:**
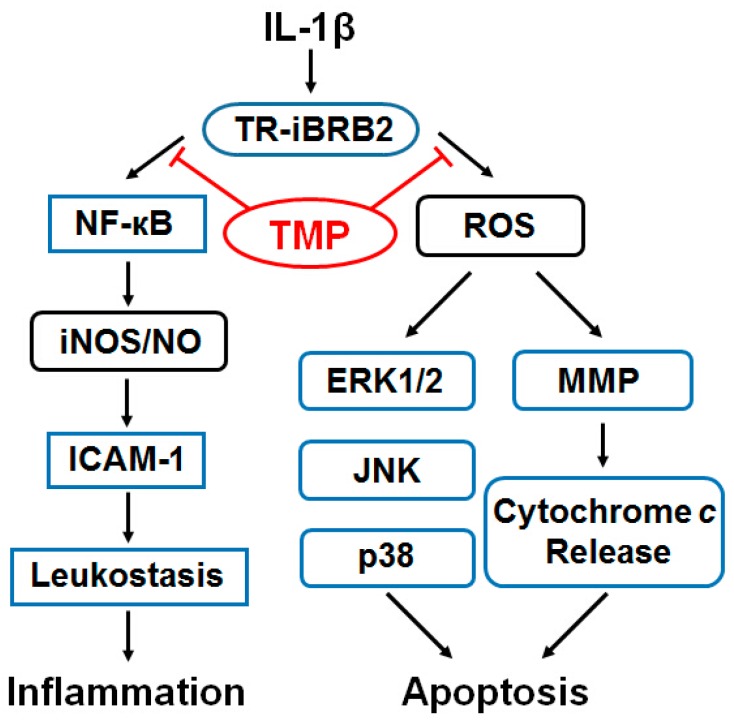
The proposed molecular mechanisms underpinning the protective effect of TMP on nitrosative and oxidative stress induced by IL-1β in retinal endothelial TR-iBRB2 cells. ROS: reactive oxygen species; iNOS: inducible nitric oxide synthase; NO: nitric oxide; MMP: mitochondrial membrane potential.

In conclusion, TMP prevented TR-iBRB2 cells from IL-1β-induced nitrative stress and oxidative stress. The molecular mechanisms underpinning the protective effect of TMP may be mediated through the inhibition of iNOS expression and leukostasis as well as suppression of ROS formation, mitochondrial dysfunction and blockade of MAPKs phosphorylation (Figure 8). Such information forms the basis of the clinical development of this compound in treating diabetic retinopathy.

## 3. Experimental Section

### 3.1. Materials

Tetramethylpyrazine was obtained from National Institute for the Control of Pharmaceutical and Biological Products (Beijing, China). Recombinant rat IL-1β was purchased from PeproTech (Rocky Hill, HJ, USA). Growth medium components were purchased from Gibco (New York, NY, USA). 3-(4,5-dimethylthiazol-2-yl)-2,5-diphenyl tetrazolium bromide (MTT), 2,7-dichlorodihydrofluorescein diacetate (DCFH-DA), Calcein AM (C-AM) and 5,5′,6,6′-tetrachloro-1,1′,3,3′-tetraethylbenzimidazolylcarbocyanine (JC-1) were purchased from Sigma (St. Louis, MO, USA). Dimethyl sulfoxide (DMSO), sodium bicarbonate, penicillin-streptomycin, trypsin, ribonuclease A (RNase A), polyvinylidenefluoride (PVDF) membrane, Griess reagent and enhanced chemiluminescence (ECL) assay kit were purchased from Beyotime (Nantong, China). Trizol reagent and RT-PCR reagent kit were purchased from Takara (Dalian, China). Annexin V-FITC and propidium iodide (PI) double staining kit was purchased from Becton-Dicknson (Franklin Lakes, NJ, USA). The information about the antibodies used in this study shows as following: CD54 (intercellular adhesion molecule-1) (MAB583; R&D System, Minneapolis, MN, USA), iNOS (ab3523; Abcam, Cambridge, MA, USA), NF-κB p65 (100-4165; Biomol, Hamburg, Germany), cytochrome *c* (556433; BD Biosciences, San Jose, CA, USA), ERK1/2 (#9102; CST, Beverly, MA, USA), JNK (#9251; CST), p38 (#9212; CST), phosphor-ERK1/2 (#9106; CST), phosphor-JNK (#9255; CST), phosphor-p38 (#9216; CST), β-actin (#4967; CST), and the HRP conjugated goat anti mouse (sc-2005; Santa Cruz, Dallas, TX, USA)/rabbit (sc-2004; Santa Cruz) secondary antibodies.

### 3.2. Cell Culture

Rat retinal endothelial cells (TR-iBRB2 cell line) were isolated from the retina of the male transgenic rats (line no. 1507-5) as previously described [14] and cultured in low-glucose DMEM containing 10% heat inactivated fetal bovine serum (FBS), 20 mM sodium bicarbonate, 15 ng/mL Endothelial cell growth factor (ECGF), 0.1 mg/mL streptomycin, and 100 IU/mL penicillin. Cells were maintained at 33 °C in a humidified atmosphere containing 5% CO_2_. For each experiment, cells were detached, re-seeded in plates and then incubated with or without drugs for the indicated time.

### 3.3. Real-Time PCR Analysis

Total RNA was isolated using Trizol reagent and first-strand cDNA was synthesized using RT reagent kit according to the manufacturer’s protocol. The primer pair sequences for amplifying iNOS and GAPDH were: iNOS, sense, 5′-TGG AGC GAG TTG TGG ATT GTC-3′, antisense, 5′- CCC TTT GTG CTG GGA GTA GT-3′; GAPDH, sense, 5′-CAA GGT CAT CCA TGA CAA CTT TG-3′, antisense, 5′-GGC CAT CCA CAG TCT TCT GG-3′. Real-time PCR was performed with the following reaction parameters: 3 min at 95 °C, 30 cycles followed for 10 s at 95 °C, 30 s at 60 °C, then 65–95 °C to dissolve. The amount of target was normalized to GAPDH (an endogenous reference) and relative to the control using the comparative ΔΔ*C*_t_ method as described before [33].

### 3.4. Western Blot Analysis

For Western blot analysis, cells (1 × 10^6^) were collected and lysed in ice-cold RIPA buffer (50 mM Tris-HCl, 150 mM NaCl, 1 mM EGTA, 1 mM EDTA, 20 mM NaF, 100 mM Na_3_VO_4_, 1% Nonidet P-40 (NP-40), 1% Triton X-100, 1 mM phenylmethylsulfonyl fluoride (PMSF), 10 mg/mL Aprotinin and 10 mg/mL Leupeptin) for 30 min. Protein concentration was determined by the Bradford method [34] and 50 μg protein from each sample was used for Western blot analysis. Protein was separated by electrophoresis on 10% sodium dodecyl sulfate-polyacrylamide gel electrophoresis (SDS-PAGE) and transferred electrophoretically to polyvinylidene difluoride (PVDF) membranes. After blocking with 5% bovine serum albumin (BSA) in the mixture of Tris-Buffered Saline and Tween-20 (TBST) for 1 h, membranes were incubated with primary antibody overnight and followed by incubation with secondary antibody for 1 h at room temperature. Protein bands were visualized using the ECL assay kit. The density of each band was normalized to the expression of β-actin.

### 3.5. NO Measurement

Nitrite (NO_2_^−^), converted from NO and molecular oxygen, indicates the extracellular NO release from activated TR-iBRB2 cells. TR-iBRB2 cells were plated in 24-well plates and grown to reach confluence overnight. After pretreatment with or without TMP at various concentrations for 12 h, cells were exposed to IL-1β (10 ng/mL) for 6 h. Then the culture medium was collected and processed for the Griess assay. The required volume of culture medium (50 μL) was mixed with an equal (1:1) volume of Griess reagent (1% sulfanilamide and 0.1% *N*-naphthylethyl-ethylenediamine dihydrochloride in 5% phosphoric acid) for 10 min at 37 °C in the dark. The absorbance was measured at 540 nm using an ELISA reader. A standard curve was generated using NaNO_2_ in each experiment.

### 3.6. Measurement of ROS

The generation of intracellular ROS was measured by flow cytometry using DCFH-DA staining. DCFH-DA is a non-fluorescent compound that can be enzymatically converted to highly fluorescent compound DCF, in the presence of ROS. After pretreatment with TMP at various concentrations for 12 h, cells were exposed to IL-1β (10 ng/mL) for 6 h. Then TR-iBRB2 cells were incubated with DCFH-DA (10 μM) for 30 min at 37 °C in dark. After washing twice with PBS, fluorescence intensity was analyzed by flow cytometry [35].

### 3.7. Leukocyte-Endothelial Cell Adhesion Assay

TR-iBRB2 cells pretreated with indicated drugs were plated at a density of 1.2 × 10^4^ cells/well in flat clear bottom black 96-well plates and cultured to confluence overnight. Rat lymphocytes were isolated with the density-gradient centrifugation and suspended in RPMI 1640 with 10% FBS and incubated with 2 mM C-AM for 30 min at 37 °C, then the cells were washed with PBS for three times to remove the free C-AM. The suspension of C-AM labeled cells (5 × 10^5^ cells/well) was added to incubate with the monolayer of TR-iBRB2 cells for 90 min at 37 °C. After incubation, non-adherent cells were removed by gentle washing with RPMI. C-AM fluorescence in the labeled cells was measured in a fluorescence plate reader at an excitation wavelength of 490 nm and an emission wavelength of 530 nm.

### 3.8. Cell Viability Assay

Cell viability was evaluated with the methyl thiazoyltetrazolium (MTT) assay. TR-iBRB2 cells were seeded at a density of 7.5 × 10^3^ cells/well in 96-well plates and cultured to confluence overnight. Briefly, cells were pretreated with TMP at concentrations ranging from 0 to 25 μM for 12 h before exposure to IL-1β (10 ng/mL) for 12 h. The cells were washed with PBS and incubated with 200 μL medium containing 20 μL of MTT (1 mg/mL) at 37 °C for 4 h. The medium was then aspirated and 150 μL of DMSO per well was added for formazan solubilization. The absorbance of converted dye was measured at a wavelength of 490 nm using a fluorescence plate reader (SpectraMax M5, Molecular Devices, Sunnyvale, CA, USA). The viability of TR-iBRB2 cells in each well was presented as percentage of control cells.

### 3.9. Cell Apoptosis Assay

Apoptosis of cells was examined by double staining with Annexin V-FITC and PI. After treatment with indicated drugs, cells were washed twice with ice-cold PBS and re-suspended in 300 μL binding buffer containing 10 μL of Annexin V-FITC stock and 10 μL of PI. After incubation for 15 min at room temperature in dark, the samples were then analyzed by flow cytometry. The Annexin V^+^/PI^−^ cells were considered as apoptotic cells and the percentage of which was calculated by CellQuest software (Becton-Dickinson, Franklin Lakes, NJ, USA).

### 3.10. Measurement of MMP

The level of MMP was determined by flow cytometry using mitochondrial-specific cationic dye JC-1. Briefly, after pretreatment with TMP at various concentrations for 12 h, the cells were exposed to IL-1β (10 ng/mL) for 6 h. Then cells were washed and incubated with JC-1 (25 μM) for 30 min at 37 °C. The level of MMP was then analyzed by flow cytometry. Fluorescence was monitored at wavelengths of 490 nm (excitation)/530 nm (emission) and 530 nm (excitation)/590 nm (emission). Changes in the ratio between the measurement at wavelengths of 590 nm (red) and 530 nm (green) fluorescence intensities indicated the alternation of MMP level.

### 3.11. Measurement of Cytochrome c Release

For measurement of cytochrome c release, the cytosol and mitochondrial fractions were prepared as described previously [36]. After treatment with the indicated drugs, mitochondrial and cytosolic fractions were extracted from the cells using Apo Alert Cell Fractionation kit (Clontech, Mountain View, CA, USA) according to the manufacturer’s instructions. The expression of cytochrome *c* was determined using a monoclonal antibody through Western blot analysis as described above.

### 3.12. Statistical Analysis

Biostatistical analyses were conducted using the SPSS 16.0 software package (IBM, Armonk, NY, USA). All experiments were repeated three times. Results of multiple experiments were expressed as mean ± SD. Statistical comparisons were made by one-way ANOVA followed by Tukey’s *post hoc* test and *p* value less than 0.05 was accepted as statistically significant.
